# Study of male–mediated gene flow across a hybrid zone in the common shrew (*Sorex araneus*) using Y chromosome

**DOI:** 10.3897/CompCytogen.v11i2.13494

**Published:** 2017-06-19

**Authors:** Andrei V. Polyakov, Viktor V. Panov

**Affiliations:** 1 Institute of Cytology and Genetics, the Siberian Branch of the Russian Academy of Sciences, Novosibirsk, Russia; 2 Institute of Systematics and Ecology of Animals, the Siberian Branch of the Russian Academy of Sciences, Novosibirsk, Russia

**Keywords:** *Sorex araneus*, phenotypic evolution, hybrid zone, gene flow, Y chromosome

## Abstract

Despite many studies, the impact of chromosome rearrangements on gene flow between chromosome races of the common shrew (*Sorex araneus* Linnaeus, 1758) remains unclear. Interracial hybrids form meiotic chromosome complexes that are associated with reduced fertility. Nevertheless comprehensive investigations of autosomal and mitochondrial markers revealed weak or no barrier to gene flow between chromosomally divergent populations.

In a narrow zone of contact between the Novosibirsk and Tomsk races hybrids are produced with extraordinarily complex configurations at meiosis I. Microsatellite markers have not revealed any barrier to gene flow, but the phenotypic differentiation between races is greater than may be expected if gene flow was unrestricted. To explore this contradiction we analyzed the distribution of the Y chromosome SNP markers within this hybrid zone. The Y chromosome variants in combination with race specific autosome complements allow backcrosses to be distinguished and their proportion among individuals within the hybrid zone to be evaluated. The balanced ratio of the Y variants observed among the pure race individuals as well as backcrosses reveals no male mediated barrier to gene flow. The impact of reproductive unfitness of backcrosses on gene flow is discussed as a possible mechanism of the preservation of race-specific morphology within the hybrid zone.

## Introduction

The common shrew (*Sorex araneus* Linnaeus, 1758) is assumed to be a promising model species for evolutionary studies because of the remarkable diversity of its karyotype. Ten chromosome arms joined together in various Robertsonian fusions form dozens of chromosome races (Wójcik et al. 2003) – “groups of geographically contiguous populations that share the same set of metacentrics and acrocentrics by descent” (Hausser et al. 1994). Ranges of the races do not overlap but parapatric races can establish geographic contacts in narrow zones of intergradation where they hybridize and produce interracial hybrids. At meiosis I of these hybrids, chromosomes form multivalents of different complexity following the pattern of arm homology. These multivalents are associated with reduced fertility of hybrids due to aberrations in chromosome pairing, recombination and segregation, which in turn may lead to germ cell death or/and generation of unbalanced gametes (Searle 1993). The decline in fertility can act as a mechanism to impede gene flow, contributing, thus, to speciation (King 1993). Nevertheless, comprehensive studies of protein, autosomal and mitochondrial DNA markers revealed weak or no divergence between chromosomally divergent populations [(Wójcik et al. 2002 (for review of previous works), Andersson et al. 2004, 2005, Jadwiszczak et al. 2006, Lundqvist et al. 2011, Moska et al. 2011, Horn et al. 2012)]. However, in some rare cases races inhabiting adjacent areas exhibit clear morphological distinction within the zones of intergradation (Chętnicki et al. 1996, Polyakov et al. 2002, Polly et al. 2013), providing an excellent opportunity to clarify the details of interracial contact.

The Novosibirsk and Tomsk races occupy the whole territory of West Siberia (Polyakov et al. 1996, 2000) and form there a hybrid zone approximately 8.5 kilometers in width (Polyakov et al. 2011). Characteristic chromosomes of the Novosibirsk race comprise six metacentric autosomes *go, hn, ik, jl, mp, qr*, whereas the Tomsk race is characterized by metacentrics *gk, hi, jl, mn* and acrocentrics *o, p, q, r*. An italicized letter of the alphabet indicates here a chromosome arm, which can either be unattached as an acrocentric or attached to another chromosome arm as a metacentric (Searle et al. 1991).

Interracial hybrids form a complex multivalent (a chain of nine chromosomes) *o/og/gk/ki/ih/hn/nm/mp/p* (Polyakov et al. 2004) that is expected to cause substantially reduced fertility compared to pure race individuals. This assumption is supported by the observation of a wide variety of chromosome pairing abnormalities in hybrid males, in which the overall proportion of cells with synaptic abnormalities was 13 times higher than in homozygotes (Borodin 2008).

The Novosibirsk and Tomsk races apparently evolved in allopatry during the last glacial maximum in Ural and Altai refugia, respectively (Polyakov et al. 2003). They are well differentiated for morphological traits (Yudin 1989) and DNA markers (Polyakov et al. 2009). Interracial differences in morphology remain significant even within the zone where races meet and hybridize (Polyakov et al. 2002, Polly et al. 2013). The estimated duration of hybridization is at least several hundreds of generations (Polyakov et al. 2011) and preservation of race-specific morphological features within the hybrid zone for such a long period would only be possible if the barrier to gene flow between populations is very strong (Polly et al. 2013). If this barrier arises due to the influence of chromosomal rearrangements on fertility of hybrids, the fecundity of these hybrids should be very low.

Surprisingly, analysis of microsatellites has revealed low level of differentiation within this hybrid zone, which implies a free flow of genes (Horn et al. 2012). This contradicts the results of morphological studies and requires an additional consideration. It is necessary to mention however, that analysis based on microsatellites may underestimate the values of differentiation because of high variability of these markers (Balloux et al. 2000).

To explore the contradiction between the microsatellites and morphology, it might be useful to re-examine the fertility of hybrids with an additional set of markers. If their reproductive potential is low enough to impede the introgression of morphological traits, then microsatellites can be considered an inappropriate marker system for such analyses. The impact of chromosome rearrangements on gene flow in this case will be proved. Otherwise, the mechanism of restriction of gene flow needs to be revised.

In order to estimate a contribution of males - hybrids F1 in reproduction we identified two variants of a new SNP marker in the Y chromosome intron UTY11 and examined their frequencies within the hybrid zone and at the adjacent territory of the Novosibirsk race. In this article we focus particularly on descendants of the hybrid males. The Y chromosome variants in combination with race specific autosome complements allow backcrosses to be distinguished, i.e. individuals that have the Y chromosome from one race together with autosome complement of another parental race. This combination can only occur if the Y chromosome is transmitted through the F1 male. This study is the first that examines the fitness of hybrids directly according to the presence of their descendants in population. All previous studies were based on the assessment of the level of meiotic aberrations and the width of the zones of introgression.

The methodological approach of the presented study was based on the following reasoning:

1. Balanced gametes in hybrids have either the full Novosibirsk or the full Tomsk complement of autosomes. Therefore, only three variants of karyotype - Novosibirsk homozygotes, Tomsk homozygotes and Novosibirsk/Tomsk heterozygotes, occur within the hybrid zone.

2. The Y chromosome does not recombine and thus its alleles retain their racial specificity.

3. A Y chromosome allele of one race can occur in another race only if it has been transmitted through the F1 male.

If the fertility of hybrids is so low that provides a barrier to gene flow, the expected number of backcrosses will be close to zero.

## Material and methods

**Figure 1.**
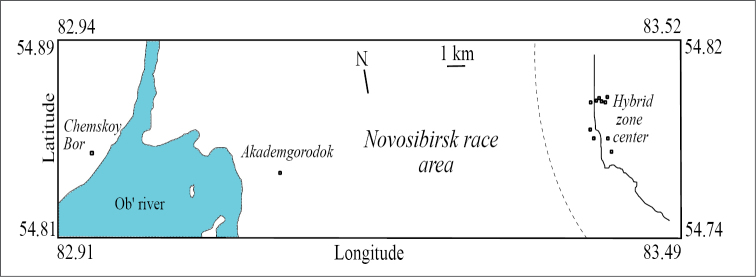
Location of sampling sites. Dotted line marks limits of introgression of the Tomsk autosomes complement; firm curved line determines the centre of the hybrid zone according to Polyakov et al. (2011).

The variability of intron UTY11 of the Y-chromosome was studied among 39 males from the centre of the hybrid zone between the Novosibirsk and Tomsk chromosome races of the common shrew (Figure 1). Of these males, 25 were homozygous for the Novosibirsk race and 14 for the Tomsk race chromosome complements. Trapping and karyotyping were performed in previous studies (Polyakov et al. 2011). Additional 32 individuals from two localities situated within the distribution range of the Novosibirsk race (27 from Akademgorodok and 5 from Chemskoy Bor) were examined. Shrews of these localities are monomorphic for the Novosibirsk race karyotypes (Král and Radjabli 1974, Polyakov et al. 1997).

Intron UTY11 of the Y chromosome was amplified following the protocol of Hellborg and Ellegren (2003). Sequencing was performed in both directions and analyzed using an ABI Prism 3100 genetic analyzer (Applied Biosystems) in the SB RAS Genomics Core Facility (Novosibirsk, Russia).

Student’s t-test statistics was used to assess the difference in the ratio of the Y haplotypes between races. The level of linkage disequilibrium between the Y haplotypes (Y) and autosome complements (A) was quantified by the coefficient of linkage disequilibrium D_YA_ = p_YA_ – p_Y_p_A_.

## Results

Two haplotypes of intron UTY11 with cytosine/thymine substitution at position 585 (C-haplotype/T-haplotype, respectively) were identified among the studied shrews (GenBank (www.ncbi.nlm.nih.gov/Genbank) accession numbers KY652093 and KY652094). Table 1 shows the distribution of these haplotypes in the studied races. The haplotype C was detected in the shrews trapped in Akademgorodok, Chemskoy Bor and in the hybrid zone. The haplotype T was detected in the hybrid zone only.

In the hybrid zone the frequency of C-haplotype (0.77) is greater than the frequency of T-haplotype (0.23), however the ratio of the Y haplotypes between shrews with the Novosibirsk and Tomsk autosome complements does not differ statistically (t_d_ = 0.59, P > 0.05).

We did not detect linkage disequilibrium between the Novosibirsk- and Tomsk- derived autosomes and the Y chromosome variants (D = 0.02, χ2 = 0.37, P > 0.05).

**Table 1.** Frequency of the Y chromosome variants in the studied races.

Localities	Autosomal complement	n	n of T-haplotype	Frequency of T-haplotype	SE
	Novosibirsk	25	5	0.20	0.08
Hybrid zone	Tomsk	14	4	0.29	0.12
	Total	39	9	0.23	0.07
Akademgorodok	Novosibirsk	27	0	0	
Chemskoy Bor	Novosibirsk	5	0	0	
	Total	32	0	0	

## Discussion

Akademgorodok and Chemskoy Bor are situated within the distribution range of the Novosibirsk race. Only the C-haplotype of the Y chromosome was found among shrews from both localities. Thus, we may suggest that the Novosibirsk race is monomorphic for this haplotype.

In the hybrid zone the frequency of C-haplotype is greater than the frequency of T-haplotype. This may indicate that both haplotypes of the Y chromosome are present in the Tomsk race. Alternatively, this could reflect a shift of the Y-chromosomal cline towards the Tomsk race area. The latter explanation is consistent with the results of previous morphological studies, where the clines in medial and lateral mandible sizes were centered at the Tomsk race side of the hybrid zone (Polly et al. 2013).

Backcrosses with the T-haplotype and autosomes of the Novosibirsk race are present in the hybrid zone. They would not be there, if the hybrid males were sterile. Nearly equal number of the T-haplotype in combination with both autosome complements implies a continuous flow of Y chromosome from the Tomsk to Novosibirsk race. This observation suggests that even if the hybrid males suffer from reduced fertility, it does not provide an insurmountable barrier to gene flow between the contacting populations.

Hybridization between divergent populations begins with the production of F1 and subsequent backcrossing. Repeated generations of backcross individuals result in introgression of mutations, collected by populations in allopatry (Maheshwari and Barbash 2011). Introgression can be prevented if hybrid incompatibilities reduce the fitness of the F1 or/and backcross generations (Turelli and Orr 2000).

Poor reproductive performance of hybrid shrews with chromosomal multivalents can be related not only to aberrations in generative tissues and gametes. The other cause can be the failure in competition for mating or low viability of their offspring. Our results indicate that none of this happens and the F1 hybrids are adequately involved in reproduction. The balanced ratio of Y variants among the pure race individuals and backcrosses in the Novosibirsk/Tomsk hybrid zone suggests that the F1 produce viable progeny. It does not explain the distinct differentiation of shrews in morphological traits. However, if this differentiation is facilitated by a barrier to gene flow, and if this barrier is determined by hybrid incompatibilities, the results of the present study make the list of possible incompatibilities shorter. Indeed, after the rehabilitation of the F1, low fertility of backcrosses remains the only thing that can be suspected to influence gene flow. Certainly, this assumption requires careful consideration. Below we discuss some issues related to the possible impact of low fertility of backcrosses on gene flow.

**Figure 2.**
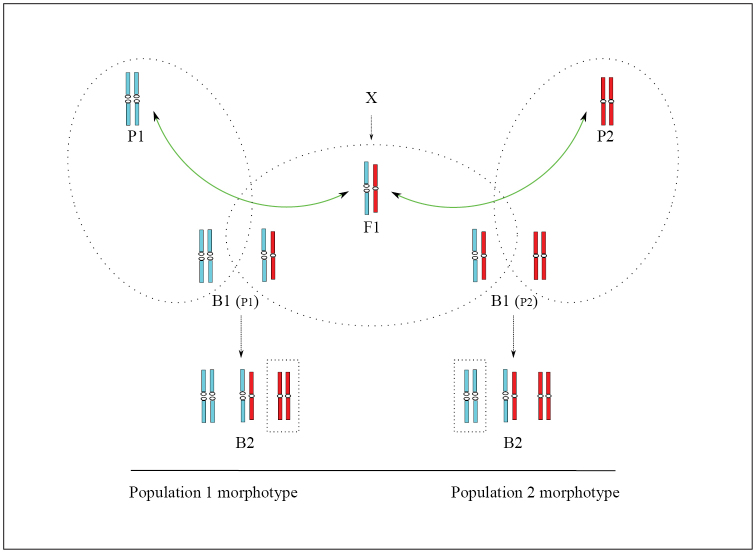
Segregation of karyotypes and morphological traits in the hybrid zone between two chromosome races. Positions of karyotypes reflect their morphological state: individuals of pure parental type (P1 and P2) with the most pronounced morphological differences occupy rightmost and leftmost positions, F1 – intermediate between P1 and P2 and the first-generation backcrosses – intermediate between F1 and respective parents (B1_P1_ and B1_P2_). The second-generation backcrosses (B2) contain karyotypes that do not correspond to the expected morphotypes (marked with squared frames). Round frames mark karyotypically indistinguishable parents, F1 and B1 (see text for details).

The inheritance of morphological traits is defined by many loci with additive effect (Kemper et al. 2012). In a study of the inheritance of body size, crosses between strains of laboratory mice with different size have been made. In these experiments the F1 and F2 means were halfway between the parents and the backcross means were halfway between the F1 and respective parents (Butler 1952, Chai 1956). Similar crosses occur among shrews within the hybrid zone. In evaluation of morphological traits of shrews with consideration of their karyotypes, all the homozygous individuals with the Novosibirsk race karyotype were significantly smaller than the Tomsk homozygous individuals (Polyakov et al. 2002). This difference enabled differentiation of two separate morphotype groups, and the Novosibirsk shrews never grouped with the Tomsk shrews and vice-versa (see figure 1 in Polyakov et al. 2002). Morphological variability of the heterozygotes was much broader and overlapped the extent of variation found in both homozygotes. Figure 2 illustrates the segregation of morphology and karyotypes in the hybrid zone as it may be expected following the experiments of Butler (1952) and Chai (1956). In this figure the relationship between the karyotypes and morphological types at stages the F1 and the first-generation backcrosses is in a good agreement with the experimental data from the hybrid zone of shrews. Parents and homozygous first-generation backcrosses form two distinct morphological groups, while karyotypically similar F1 and heterozygous first-generation backcrosses show variation that overlaps both homozygous groups. The appearance of the second-generation backcrosses, that combine the homozygous karyotypes of one race with a morphotype of the other race, would bring discrepancy in this concordance. However, no discrepancy between the karyotypes and morphological types was observed in experimental studies of the Novosibirsk/Tomsk hybrid zone, and it can be assumed that the second-generation backcrosses do not appear in this case. The reason of the absence of the second-generation backcrosses is difficult to explain unequivocally. We can only assume that if the first-generation backcrosses had been involved in reproduction, unlimited introgression could have been expected: foreign alleles would have accumulated on both sides of the hybrid zone and phenotypic differences would have become blurred after several generations. However, although hybridization between the Novosibirsk and Tomsk races has been lasting for much longer than several generations, none of this has happened.

Even a strong barrier to gene flow, based on low fertility of backcrosses, is not incompatible with the lack of differentiation of the autosomal markers including microsatellites. The populations in contact may have clear differentiation for these markers outside of the hybrid zone. If the sampling is carried out in the zone of hybridization, backcrosses will be collected together with pure race specimens. Recombination in the F1 shuffles mutations between the race specific chromosome complements and backcrosses inherit alleles from both races. The karyotypes of homozygous backcrosses are indistinguishable from the karyotypes of the pure race individuals. Appearance of these backcrosses in the same group with the pure race individuals may significantly reduce the observed differentiation. Evaluation of samples collected within the zone of hybridization may thus explain the failure of previous studies to demonstrate a distinct differentiation.

Low reproductive ability of the generation following the F1 can become a promising hypothesis for further studies of barriers to gene flow between the chromosome races of the common shrew.

## Conclusion

Aberrations in pairing, recombination and segregation of chromosomes in hybrids with complex meiotic configurations are a generally assumed barrier to gene flow among the karyotypically divergent chromosome races of the common shrew (Searle 1993). The presented study suggests that gene incompatibilities in backcrosses may have more substantial influence on gene flow than erroneous behaviour of chromosomes at meiosis. Apparently, the Novosibirsk and Tomsk races have not yet reached the final stage of divergence, when hybridization does not go beyond the F1 production; however, the poor reproductive performance of the first-generation backcrosses may preserve the adaptive genetic architecture from assimilation and thus contribute to further divergence, promoting the progress of speciation.

## References

[B1] Andersson A-C Narain Y Tegelström H Fredga K (2004) No apparent reduction of gene flow in a hybrid zone between the West and North European karyotypic groups of the common shrew, *Sorex araneus*. Molecular Ecology 13: 1205–1215. https://doi.org/10.1111/j.1365-294X.2004.02146.x 15078456 10.1111/j.1365-294X.2004.02146.x

[B2] Andersson A-C Alström-Rapaport C Fredga K (2005) Lack of mitochondrial DNA divergence between chromosome races of the common shrew, *Sorex araneus*, in Sweden. Implications for interpreting chromosomal evolution and colonization history. Molecular Ecology 14: 2703–2716. https://doi.org/10.1111/j.1365-294X.2005.02584.x 16029472 10.1111/j.1365-294X.2005.02584.x

[B3] Balloux F Brünner H Lugon-Moulin N Hausser J Goudet J (2000) Microsatellites can be misleading: an empirical and simulation study. Evolution 54(4): 1414–1422. https://doi.org/10.1111/j.0014-3820.2000.tb00573.x 11005307 10.1111/j.0014-3820.2000.tb00573.x

[B4] Borodin P (2008) Chromosomes and Speciation. In: Dobretsov N Kolchanov N Rozanov A Zavarzin G (Eds) Biosphere Origin and Evolution. Springer, US, 315–325. https://doi.org/10.1007/978-0-387-68656-1_23

[B5] Butler L (1952) A study of size inheritance in the house mouse. Canadian Journal of Zoology 30: 154–171. https://doi.org/10.1139/z52-014 10.1139/cjr50d-003 24536997

[B6] Chai CK (1956) Analysis of quantitative inheritance of body size in mice. II. Gene action and segregation. Genetics 41: 165–178. 17247618 10.1093/genetics/41.2.165 PMC1209772

[B7] Chętnicki W Fedyk S Banaszek A Szalaj K Ratkiewicz M (1996) Morphometrical characteristic of the common shrew (*Sorex araneus* L.) from interracial hybrid zones. Hereditas 125: 201–207. https://doi.org/10.1111/j.1601-5223.1996.00201.x

[B8] Hausser J Fedyk S Fredga K Searle JB Volobouev VT Wójcik JM Zima J (1994) Definition and nomenclature of chromosome races of Sorex araneus. Folia Zoologica 43(1): 1–9.

[B9] Hellborg L Ellegren H (2003) Y chromosome conserved anchored tagged sequences (YCATS) for the analysis of mammalian male specific DNA. Molecular Ecology 12: 283–291. https://doi.org/10.1046/j.1365-294X.2003.01702.x 12492896 10.1046/j.1365-294x.2003.01702.x

[B10] Horn A Basset P Yannic G Banaszek A Borodin PM Bulatova NS et al (2012) Chromosomal rearrangements do not seem to affect the gene flow in hybrid zones between karyotypic races of the common shrew (*Sorex araneus*). Evolution 66(3): 882–889. https://doi.org/10.1111/j.1558-5646.2011.01478.x 22380446 10.1111/j.1558-5646.2011.01478.x

[B11] Jadwiszczak K Ratkiewicz M Banaszek A (2006) Analysis of molecular differentiation in a hybrid zone between chromosomally distinct races of the common shrew Sorex araneus (Insectivora: Soricidae) suggests their common ancestry. Biological Journal of the Linnean Society 89: 79–90. https://doi.org/10.1111/j.1095-8312.2006.00659.x

[B12] Kemper KE Visscher PM Goddard ME (2012) Genetic architecture of body size in mammals. Genome Biology 13(4): 244. https://doi.org/10.1186/gb-2012-13-4-244 10.1186/gb-2012-13-4-244 PMC3446298 22546202

[B13] King M (1993) Species evolution: the role of chromosome change. Cambridge University Press, Cambridge, 322 pp.

[B14] Král B Radjabli SI (1974) Banding patterns and Robertsonian fusion in the Western Siberian population of *Sorex araneus* (Insectivora, Soricidae). Folia Zoologica 23: 217–227.

[B15] Lundqvist A-C Alström-Rapaport C Tegelström H (2011) Fennoscandian phylogeography of the common shrew *Sorex araneus*. Postglacial recolonization-combining information from chromosomal variation with mitochondrial DNA data. Acta Theriologica 56: 103–116. https://doi.org/10.1007/s13364-010-0022-9

[B16] Maheshwari S Barbash DA (2011) The genetics of hybrid incompatibilities. Annual Review of Genetics 45: 331–355. https://doi.org/10.1146/annurev-genet-110410-132514 10.1146/annurev-genet-110410-132514 21910629

[B17] Moska M Wierzbicki H Macierzyńska A Strzała T Maślak R Warchałowski M (2011) A microsatellite study in the Łegucki Młyn/Popielno hybrid zone reveals no genetic differentiation between two chromosome races of the common shrew (*Sorex araneus*). Acta Theriologica 56: 117–122. https://doi.org/10.1007/s13364-011-0029-x 21475705 10.1007/s13364-011-0029-x PMC3061409

[B18] Polly PD Polyakov AV Ilyashenko VB Onischenko SS White TA Shchipanov NA et al (2013) Phenotypic variation across chromosomal hybrid zones of the common shrew (*Sorex araneus*) indicates reduced gene flow. PLOS ONE 8(7): e67455. https://doi.org/10.1371/journal.pone.0067455 10.1371/journal.pone.0067455 PMC3707902 23874420

[B19] Polyakov AV Volobouev VT Borodin PM Searle JB (1996) Karyotypic races of the common shrew (Sorex araneus) with exceptionally large ranges: the Novosibirsk and Tomsk races of Siberia. Hereditas 125: 109–115. https://doi.org/10.1111/j.1601-5223.1996.00109.x

[B20] Polyakov AV Chadova NB Rodionova MI Panov VV Dobrotvorsky AK Searle JB et al. (1997) Novosibirsk revisited 24 years on: chromosome polymorphism in the Novosibirsk population of the common shrew *Sorex araneus* L. Heredity 79: 172–177. https://doi.org/10.1038/hdy.1997.140 9279011 10.1038/hdy.1997.140

[B21] Polyakov AV Zima J Banaszek A Searle JB Borodin PM (2000) New chromosome races of the common shrew *Sorex araneus* from Eastern Siberia. Acta Theriologica 45(1): 11–18. https://doi.org/10.4098/AT.arch.00-57

[B22] Polyakov AV Onischenko SS Ilyashenko VB Searle JB Borodin PM (2002) Morphometric difference between the Novosibirsk and Tomsk chromosome races of *Sorex araneus* in a zone of parapatry. Acta Theriologica 47: 381–387. https://doi.org/10.1007/BF03192464

[B23] Polyakov AV Volobouev VT Aniskin VM Zima J Searle JB Borodin PM (2003) Altitudinal partitioning of two chromosome races of the common shrew (*Sorex araneus*) in West Siberia. Mammalia 67: 201–207. araneus) in West Siberia. Mammalia 67: 201–207. https://doi.org/10.1515/mamm.2003.67.2.201

[B24] Polyakov AV Bukina MS Borodin PM (2004) Karyotype evolution and formation of mammals diversity. Contemporary problems of ecology 5: 629–634. [In Russian]

[B25] Polyakov AV Ilyashenko VB Onischenko SS Panov VV Borodin PM (2009) AFLP diversity between the Novosibirsk and Tomsk chromosome races of the common shrew (*Sorex araneus*). Comparative Cytogenetics 3: 85–89. https://doi.org/10.3897/compcytogen.v3i2.14

[B26] Polyakov AV White TA Jones RM Borodin PM Searle JB (2011) Natural hybridization between extremely divergent chromosomal races of the common shrew (*Sorex araneus*, Soricidae, Soricomorpha): hybrid zone in Siberia. Journal of Evolutionary Biology 24(7): 1393–1402. https://doi.org/10.1111/j.1420-9101.2011.02266.x 21507114 10.1111/j.1420-9101.2011.02266.x

[B27] Searle JB Fedyk S Fredga K Hausser J Volobouev V (1991) Nomenclature for the chromosomes of the common shrew (Sorex araneus). Mémoires de la Société Vaudoise des Sciences Naturelles 19: 13–22.

[B28] Searle JB (1993) Chromosomal hybrid zones in eutherian mammals. In: Harrison RG (Ed.) Hybrid Zones and Evolutionary Process. Oxford University Press, New York, 309–353.

[B29] Turelli M Orr HA (2000) Dominance, epistasis and the genetics of postzygotic isolation. Genetics 154: 1663–1679. 10747061 10.1093/genetics/154.4.1663 PMC1461023

[B30] Wójcik J Ratkiewicz M Searle JB (2002) Evolution of the common shrew *Sorex araneus*: chromosomal and molecular aspects. In: Gliwicz J (Ed. ) Theriology at the turn of a new century, Acta Theriologica 47(1): 139–167.

[B31] Wójcik J Borodin PM Fedyk S Fredga K Hausser J Mishta A et al. (2003) The list of the chromosome races of the common shrew *Sorex araneus* (updated 2002). Mammalia 67: 169–178. https://doi.org/10.1515/mamm.2003.67.2.169

[B32] Yudin BS (1989) [Insectivorous mammals of Siberia]. Nauka, Novosibirsk, 360 pp. [In Russian]

